# Mechanical stress-induced cell death in breast cancer cells

**DOI:** 10.1242/bio.043133

**Published:** 2019-08-07

**Authors:** Satomi Takao, Minoru Taya, Cerwyn Chiew

**Affiliations:** Department of Mechanical Engineering, University of Washington, Seattle, WA 98195, USA

**Keywords:** Oscillating compression stress, Breast cancer cells, Apoptosis, Necrosis, Mechanical stress-induced cell death (MSICD)

## Abstract

Providing an external mechanical stress to cancer cells seems to be an effective approach to treat cancer locally. Numbers of reports on cancer cell death subjected to mechanical stress loading are increasing, but they are more focused on apoptosis. Inducing necrosis is also important in attracting more immune cells to the cancer site via the release of danger-associated molecular patterns from cancer cells. Here we applied dynamic compression to breast cancer cells with a low frequency (0.1–30 Hz) and for a short duration (30–300 s) and they resulted in a mixed mode of apoptosis and necrosis dominant with necrotic cell death, which we call mechanical stress-induced cell death (MSICD). The necrotic cell damage of mechanically treated breast cancer cells increased in a force-dependent and time-dependent manner while a trend of frequency-independent MSICD was observed.

## Abstract (precis)

**Summary:** Dynamic mechanical compressive stress loading applied to two different breast cancer cell lines results in mechanical stress-induced cell damage (MSICD) composed of apoptosis and necrosis.

## INTRODUCTION

Various types of stress can trigger cells to undergo apoptotic and/or necrotic cell death and many researchers have claimed that mechanical stress (MS) was one of the contributors to this event (Mayr et al., 2000; Cheng et al., 2009; Kong et al., 2013; Lien et al., 2013; Gao et al., 2014; Andarawis-Puri et al., 2014). These MS varied from static stress (normal compression and shear stress) to cyclic stress loading. It is still unclear what threshold of these MS and what mode of MS loading under which type of cell death takes place. In this article, we will review the previous works related to MS-induced apoptosis and/or necrosis in cancer cells. The Jain group reported cell damage under quasi-static compression loading; they found (i) an increase in the apoptosis of cancer cells is observed under increasing MS when studying the effects of quasi-static MS on the growth and proliferation of murine mammary carcinoma cell lines (67NR and EMT6) (Cheng et al., 2009); (ii) excessive stresses (>5.8 mmHg) triggered apoptosis and impeded cell migration (Tse et al., 2012); (iii) the stress-induced growth inhibition of plateau-phase spheroids is accompanied by decreased apoptosis (Helmlinger et al., 1997); and (iv) the growth-induced solid stress by the quasi-static compression leads to hypoxia, promotes tumor progression, immunosuppression and thus, lowers the efficacy of chemo-, radio- and immunotherapies (Stylianopoulos et al., 2012). These studies imply that the use of quasi-static compression stress, even applied externally, may not be an effective approach to induce apoptosis in cancer cells.

Lien et al. (2013) reported *in vitro* experiments on the role of static laminar shear stress and oscillatory shear stress on the apoptosis of four different human cancer cell lines (Hep3B hepatocarcinoma cells, MG63 osteosarcoma cells, SCC25 oral squamous cells and A549 carcinomic alveolar basal epithelial cells) and concluded that static laminar shear stress resulted in apoptosis of cancer cells, while oscillatory (or dynamic) shear stress did not contribute in cell death.

The Ueno group (Ogiue-Ikeda et al., 2004; Yamaguchi et al., 2005, 2006) studied cell damage under a magnetic field with magnetizable beads (overall size is 4.5 µm) or under combined use of an anti-cancer drug and found: (i) aggregated cell/bead/antibody complexes can destruct targeted TCC-S leukemic cells under pulsed magnetic force (monophasic pluses of 150 µs for electric current, but corresponding to 25 Hz of magnetic field oscillations) with magnetic flux density of 2.4 tesla (T) (Ogiue-Ikeda et al., 2004); (ii) a 62% decrease in tumor weight in an *in vivo* mouse experiment ­– the effectiveness of cancer suppression was shown by dynamic magnetic pulsation by applying magnetic pulses of lower magnitude (25 pulses/s, 0.25 T) (Yamaguchi et al., 2005); and (iii) the viability of cells is much reduced under the combined use of both magnetic pulsation and the anti-cancer drug, based on an *in vivo* experiment using mice and applying both repetitive pulsed magnetic stimulation (0.25 T and frequency of 25 pulses/s for up to 6000 pulses) and imatinib on TCC-S cells (Yamaguchi et al., 2006).

Domenech et al. (2013) used iron oxide magnetic nanoparticles conjugated with epidermal growth factor receptors, which are taken up into endosomes and lysosomes due to receptor-mediated endocytosis of the target reception, thus suppressing cancer cell growth effectively under an alternating current (AC) magnetic field of 233 kHz, where the use of such a higher frequency is expected to induce a temperature rise in the cells, which is considered as hyperthermia-based apoptosis of cancer cells. Zhang et al. (2014) performed an *in vivo* experiment, inducing apoptosis in rat insulinoma tumor cells and human pancreatic beta cells by using super paramagnetic iron oxide nanoparticles (SPION) conjugated with antibodies targeting the lysosomal protein marker LAMP1 (LAMP1-SPION) where LAMP1-SPIONs are forced to spin about their own axis under the applied magnetic field with a modest frequency of 20 Hz. Similarly, several groups are using the spinning motions of micron-sized discs at relatively low frequencies (10–50 Hz) under an applied rotational magnetic field to induce apoptotic cell death of cancer cell lines (N10 human glioblastoma, SKRC-59 human renal carcinoma cells) (Kim et al., 2010; Leulmi et al., 2015). The above spinning motions of nanoparticles and micron-sized discs are considered to provide mainly a shear stressing mode to target cancer cells, resulting in apoptosis of the target cancer cells. Under an applied magnetic field of 90 Oe at a frequency of 20 Hz, cancer cells seem to be killed with more necrosis mode (∼90% necrosis versus ∼60% apoptosis) (Kim et al., 2010).

The above literature survey reveals that the use of dynamic normal stress, shear stress or a combination of them on the small area of cancer cells may be a new effective approach to induce apoptotic cell death. As such, narrowly applied MS loading signals would rapidly propagate through the cytoskeleton network reaching the site of the nucleus, thus damaging DNA and mitochondria structures (Wang et al., 2009), which is a key process of apoptosis of cells. By the approach similar to this mechanism, Tomasini et al. (2010) used a molecular dynamics model to predict the rupture mode of cell membranes made of lipid bilayers to conclude that the rupture of the cell membrane takes place under both tension and shear loading, with the shear mode being more injurious.

From the above literature survey, it is clear that no study has been reported yet on the oscillating compression stress loading on cancer cells, particularly at lower frequencies and also that the majority of the above studies on MS-induced cell death (MSICD) of various cancer cells are focused on the apoptotic cell mode of cancer cells and do not discuss cell death by necrosis as much, nor the combined mode of apoptosis and necrosis of cancer cells under MS loading. To attract more immune cells to the cancer site, necrosis also plays an important role by releasing danger-associated molecular patterns in tumor microenvironment (Kroemer et al., 2013).

This paper focuses on the MSICD mechanisms involving both necrosis and apoptosis of two breast cancer cell lines (BT-474 and MDA-MB-231) under applied oscillatory compressive MS loading at low frequencies.

## RESULTS

**Fig. 1.**
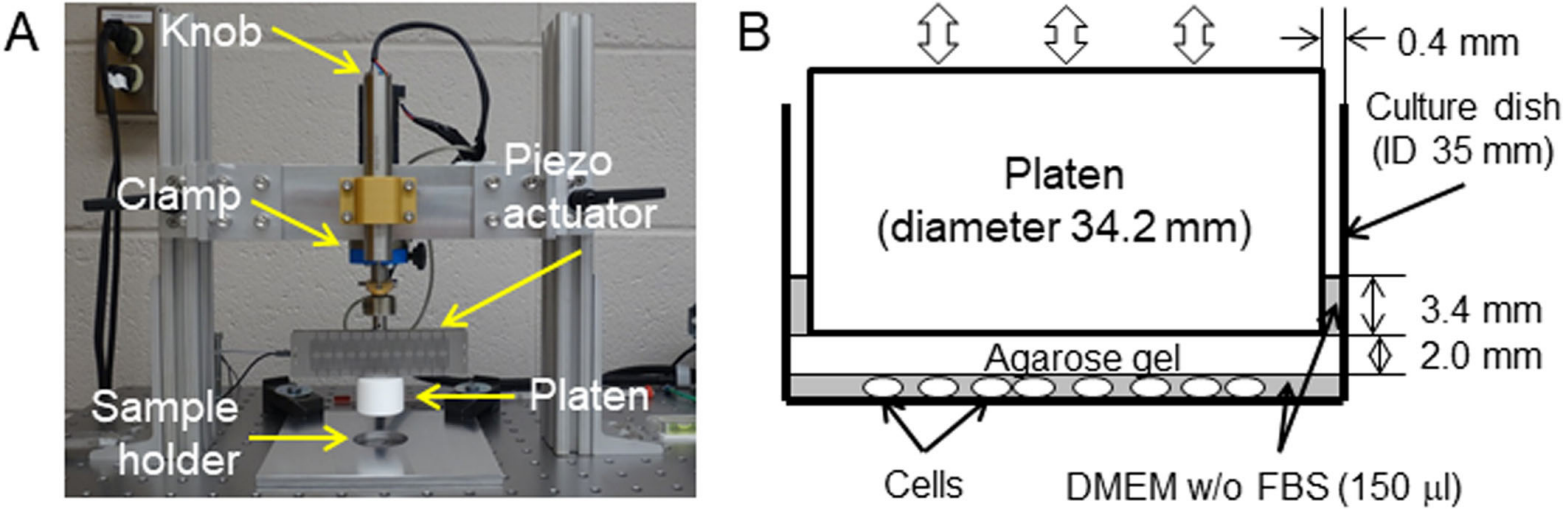
**Experimental setup for MSICD study.** (A) Mechanical stress loading device. (B) An experimental setup for sample holder with cells.

**Fig. 2.**
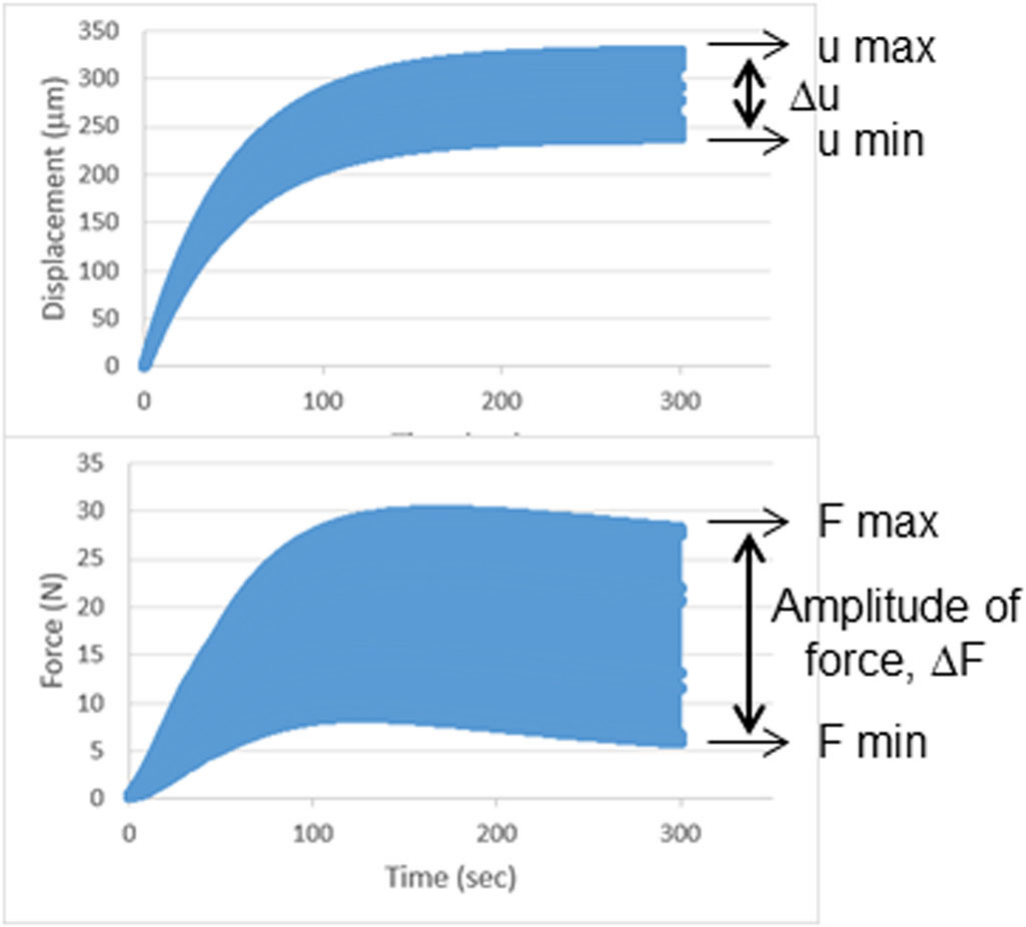
**Output data for displacement and force applied to cells.** (Top) Graph of typical displacement-time loading curve. (Bottom) Graph of the resulting force-time curve, where the amplitude of the force is given as Δ*F*=*F*_max_−*F*_min_.

**Fig. 3.**
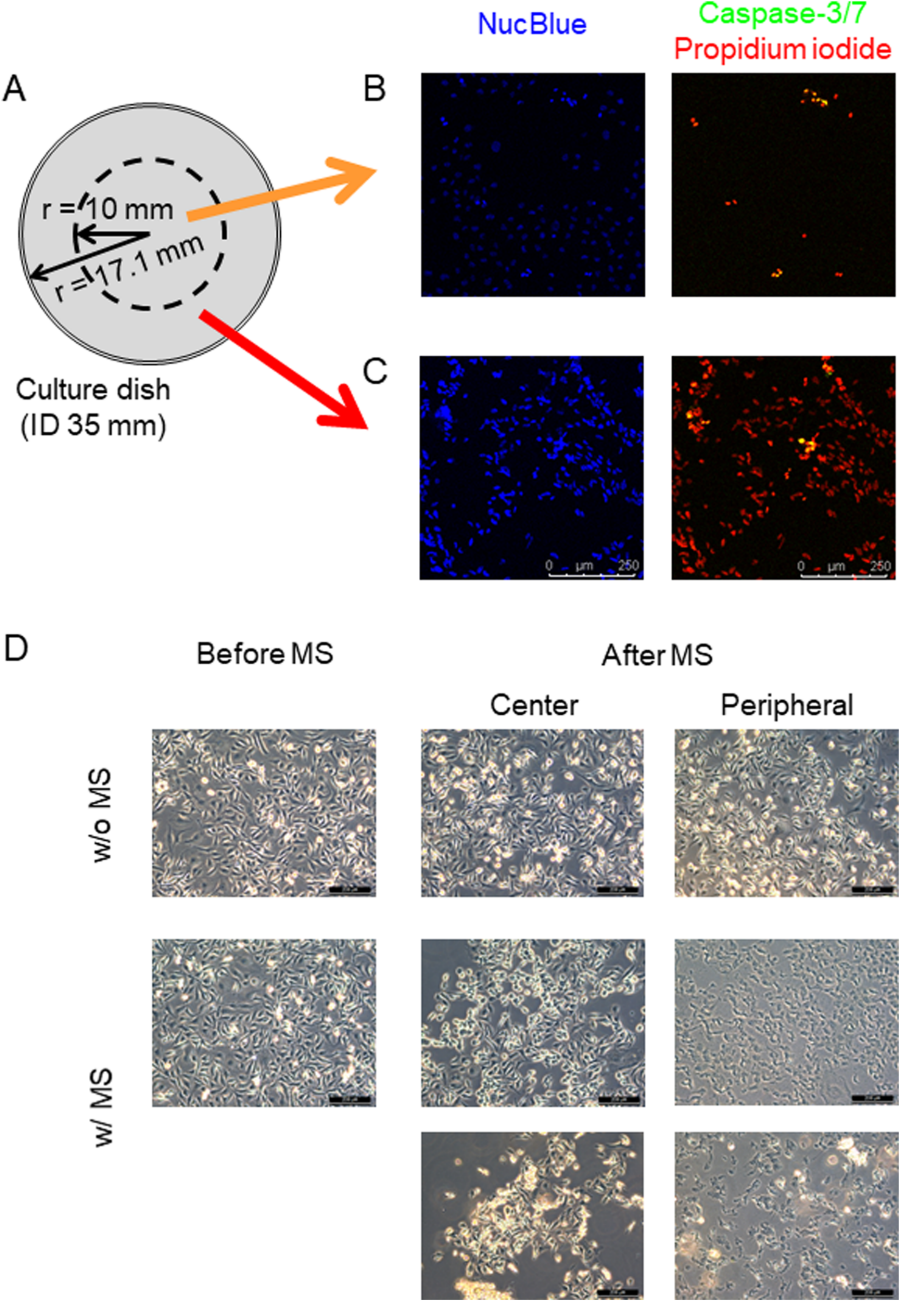
**MSICD in two regions.** (A) Dimensions of two regions; the center (radius, 0<r<10 mm) and the peripheral region (radius, 10 mm<r<17.1 mm). (B,C) MDA-MB-231 cells showed a mixed mode of apoptosis and necrosis in the central (B) region. (C) MDA-MB-231 cells with necrosis dominant in the peripheral (C) region. Cells were stained with NucBlue, Caspase-3/7 Green probe and Propidium Iodide. Green and red colors indicate the cell damage associated with apoptosis and necrosis, respectively. Scale bars: 250 µm. (D) Cell morphology change in MDA-MB-231 after MS was applied (9.3 kPa, 300 s). Control treatment group ‘w/o MS’ is shown for comparison. Scale bars: 200 µm.

We applied dynamic MS loading to breast cancer cells by using a homemade apparatus (Fig. 1) and induced apoptotic and necrotic cell death, with necrosis dominant, in those cells. The applied force amplitude (ΔF) was 3.6–19.5 N and 0.1–1.0 N on average by controlling the displacement α setting at 40–130 µm (Experiment 1) and 10 µm (Experiment 2), respectively, in the program (see Fig. 2). We subcategorized the area of culture dish in two regions as shown in Fig. 3A, where the center region (0 mm≤r<10 mm) consisted of a mixture of apoptosis and necrosis and the peripheral region (10 mm≤r≤17.1 mm) was occupied with more necrotic cells (Fig. 3B,C). When the cells were treated with a larger displacement (α) and for a relatively longer duration they were compressed, but the cell shape was still round in the central region. However, many cells in the peripheral region seem to be stretched out into an elongated spheroidal shape (Fig. 3D).

**Fig. 4.**
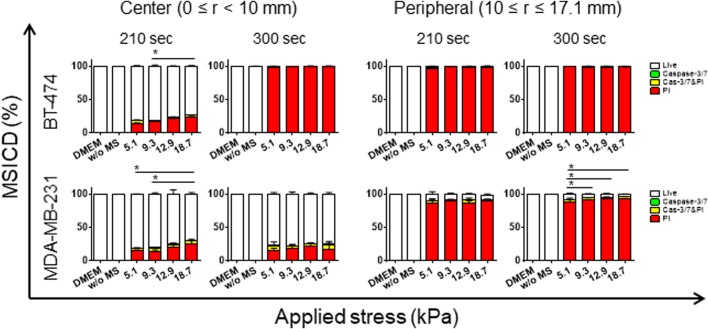
**MSICD in two breast cancer cell lines with larger displacement (equivalent stress loading) and longer duration (Experiment 1).** MSICD are plotted as a function of applied stress along the horizontal axis for different times (*t*=210, 300 s), where the upper and lower data groups are for BT-474 and MDA-MB-231, respectively. Data shown as mean±s.d. (*n*=3). *P<0.05.

**Fig. 5.**
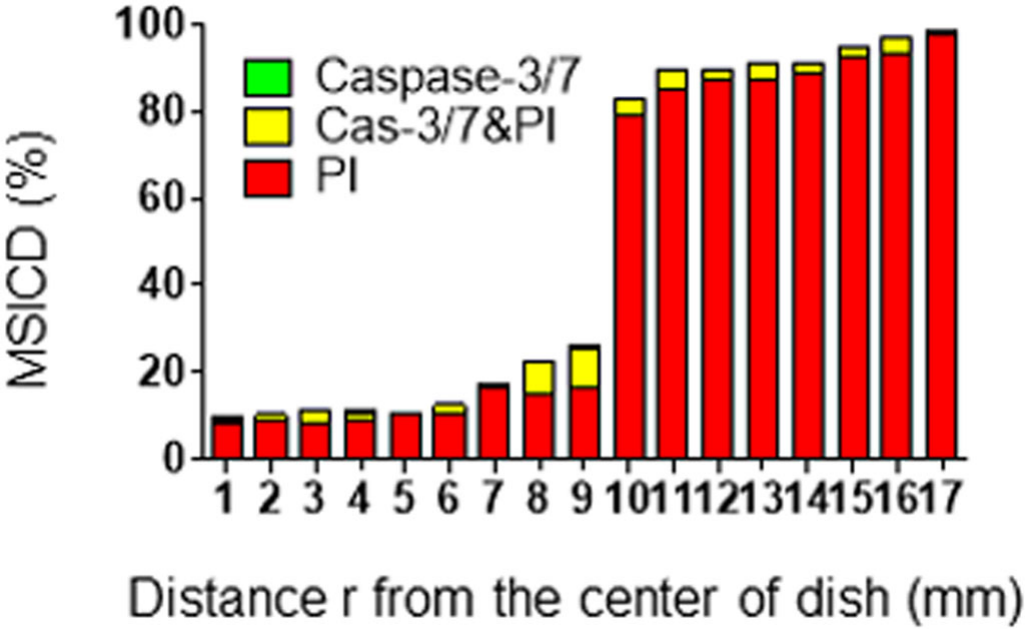
**Correlation between radius of culture dish and the rate of MSICD in MDA-MB-231.** Cells were treated for 300 s with 40 µm displacement loading. Radius is measured from the center of the petri dish toward the radial direction.

In Experiment 1, a very small number of cells in the DMEM or without MS groups showed caspase-3/7 activity (0–0.2%) and loss of cell membrane integrity (0–0.9%), indicating apoptosis and necrosis in cells in both regions, respectively, whereas MS-loaded cells resulted in a much higher rate of necrotic cell death (*P*<0.05) with a slight increase of apoptosis in both regions for the two cell lines tested (Fig. 4A,B). Displacement loading of α=40, 70, 100 and 130 µm resulted in applied force magnitude of ΔF of 4.7, 8.6, 11.8 and 17.2 N, respectively, and they were converted to applied stress of 5.1, 9.3, 12.9 and 18.7 kPa, respectively. We observed a time- and force-dependent increase of MSICD in both cell lines (*P*<0.05). Among MDA-MB-231 specimens treated with 40 µm of displacement for 300 s, the MSICD rate dramatically increased with the distance (r defined in Fig. 3A) from the center of the culture dish (Fig. 5).

**Fig. 6.**
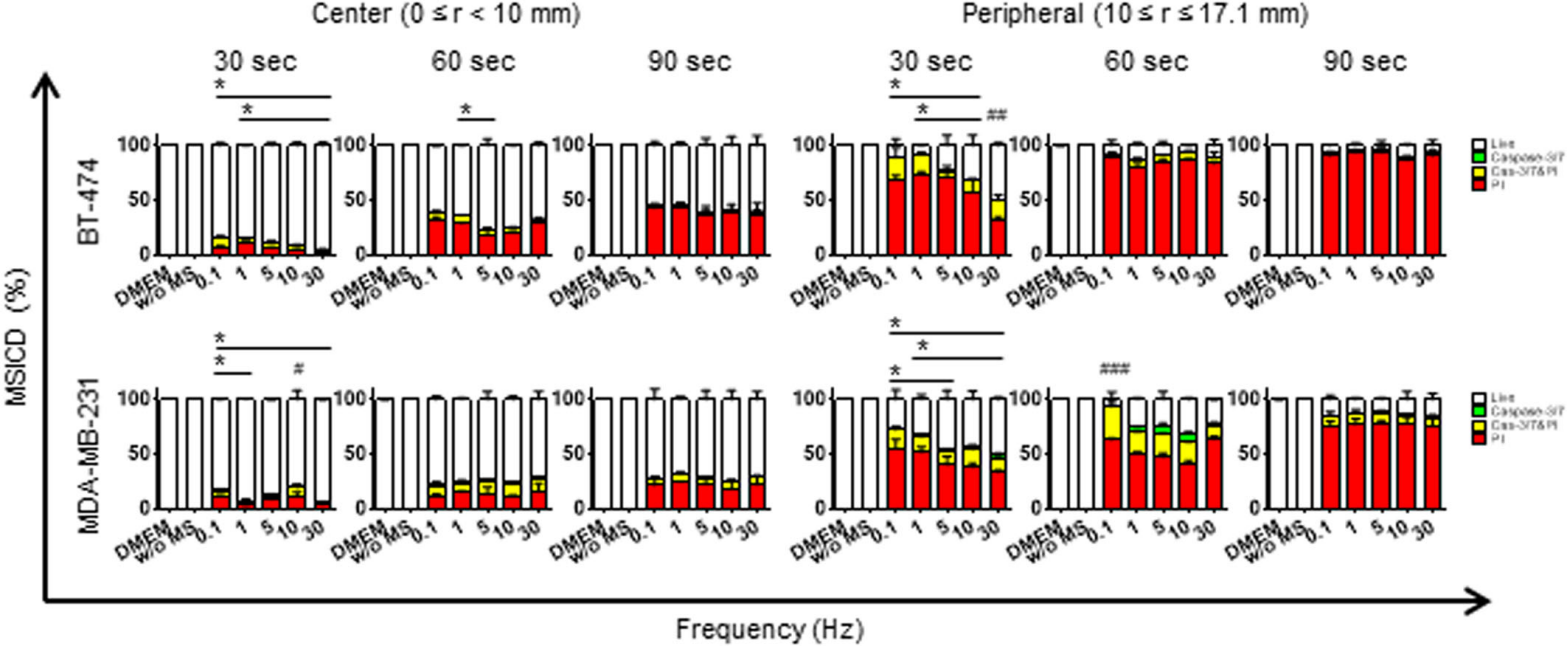
**MSCID in two breast cancer cells with smaller displacement loading (α=10** **µm) and shorter duration (Experiment 2).** MSICD are plotted as a function of frequency applied along the horizontal axis for different times (*t*=30, 60, 90 s), where the upper and lower data groups are for BT-474 and MDA-MB-231, respectively. Data shown as mean±s.d. (*n*=3).

**Fig. 7.**
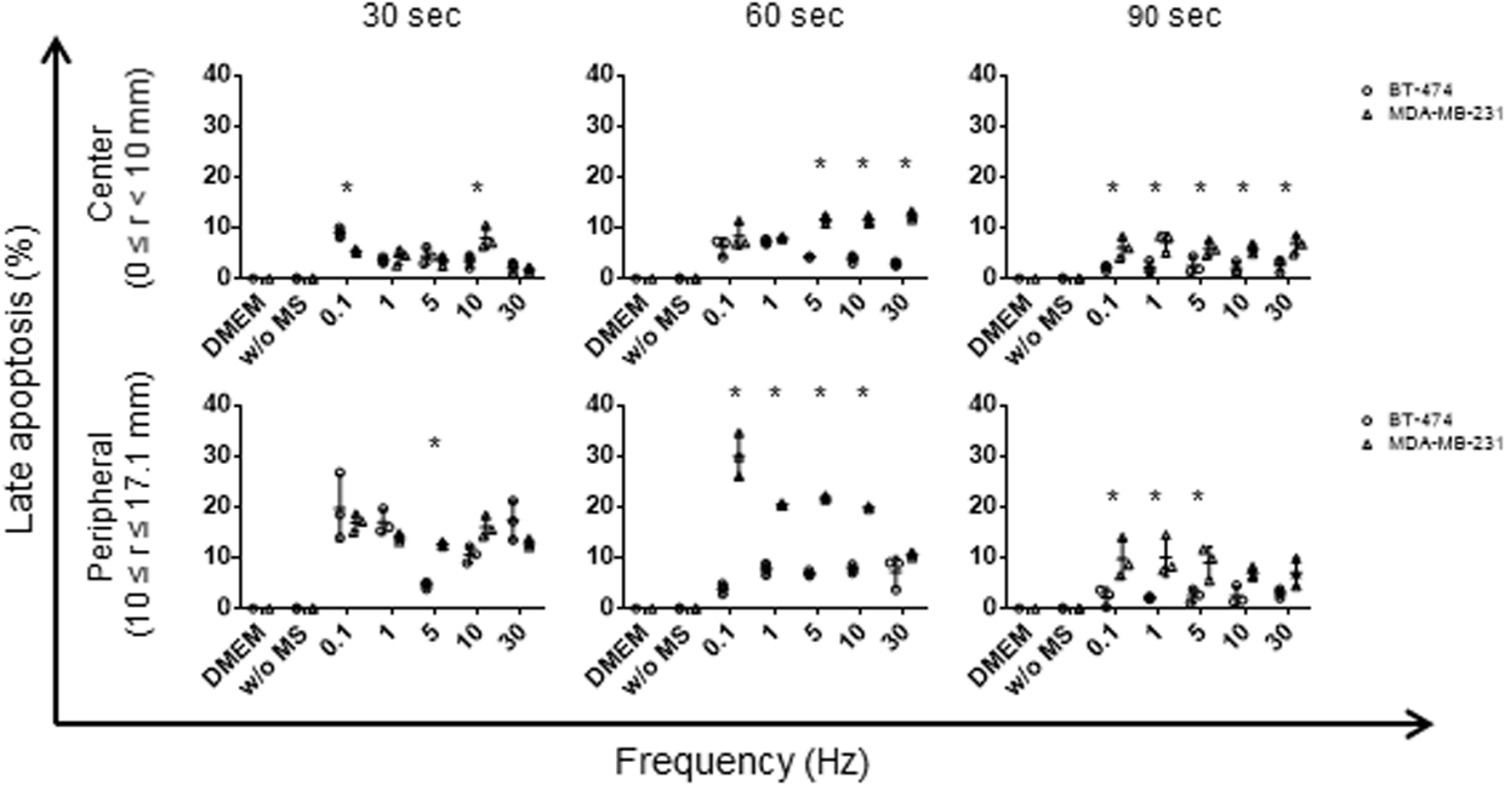
**Late apoptosis in two breast cancer cells with smaller displacement loading (α=10** **µm) and shorter duration (Experiment 2).** Late apoptosis are plotted as a function of frequency applied along the horizontal axis for different times (*t*=30, 60, 90 s), where the upper and lower data groups are for center and peripheral regions, respectively. Data shown as mean±s.d. (*n*=3). *P<0.05.

When the cells were treated with a smaller displacement loading (α=10 µm) and shorter duration in Experiment 2, limited number of cells underwent MSICD in the Dulbecco's modified Eagle Medium (DMEM) and without MS groups (0–0.1%) while MS-treated cells showed both apoptosis and necrosis. They were still necrosis-dominant but more late apoptotic cells were observed compared to Experiment 1, especially in MDA-MB-231 cells (Fig. 6, lower graphs) (*P*<0.05). The rate of MSICD was higher at 60 s and 90 s compared to that at 30 s (Fig. 6). Fig. 6 reveals a trend of frequency-dependent decreased MSICD. When two cell lines were compared, BT-474 was more sensitive to dynamic cyclic compression at a longer duration (Fig. 4) (*P*<0.05). When two cell lines were compared at a small displacement α (10 µm), MDA-MB-231 revealed more late apoptosis than BT-474 under dynamic cyclic compression, especially in the peripheral region (Fig. 7) (*P*<0.05).

## DISCUSSION

We applied a moderate frequency (0.1–30 Hz) of dynamic mechanical compression stress to breast cancer cells macroscopically by using our homemade apparatus and these cells resulted in a mixed mode of apoptosis and necrosis and they were dominantly necrotic. We named this type of cell damage MS-induced cell death (MSICD). To reduce the trauma association with cell dissociation, we specifically chose the microscopic observation to detect fluorescence signals for apoptosis and necrosis as a readout to see the effect of cyclic compression on cells. Interestingly, for two regions (center and peripheral) (Fig. 3), the former was observed as a mixture of apoptosis and necrosis and the latter was necrosis-dominant, with the shape of some cells stretched out into an elongated spheroidal shape when the MS was applied for a larger α and a relatively longer duration. We think that this phenomenon is due to the difference in stress mode; the center shows cyclic compression and the peripheral shows both cyclic compression and shear stress.

In Experiment 1, we found that the majority of cells showed necrotic damage; MSICD in necrosis mode increased with a force- and time-dependent manner in both BT-474 and MDA-MB-231 cells, while the MSICD in apoptosis mode does not exhibit similar trend (Fig. 4). We used a similar frequency (30 Hz) comparable to those used by the other researchers (Zhang et al., 2014; Kim et al., 2010; Leulmi et al., 2015). We first tested with longer durations, such as 30 min, but all cells showed necrosis even for the small stress of ∼5 kPa (data not shown). Thus we set the time much shorter, but still necrosis occurred at >90% by 300 s in both cell lines. In the study by Kim et al. (2010), human glioblastoma cells resulted in 90% of lactate dehydrogenase leakage (rupture of cell membrane) and 60% of DNA fragmentation (late apoptosis), by using magnetic-vortex microdiscs when 20 Hz of magnetic field was applied for 10 min, which shows a similar rate of necrotic cell damage to ours.

Compared to necrosis, there was no clear trend of force- or time-dependent increase of apoptosis in both cell lines (green and yellow, Fig. 4). MSCID in apoptosis mode was much less observed with the highest rate of 12.7%. Caspase 3 and 7 are proteins that execute the process of apoptosis and the probe we used can detect early stage of apoptosis. Necrosis-dominant cell death in our case could be explained by the range of α amplitude (40–130 µm) and force (4.7–17.2 N) we applied to the cells. The smallest α (40 µm) was larger than the typical height of cells and the range of applied force was much larger than the force needed to rupture a cell membrane (hundreds of pN) (Müller et al., 2015). The stress we applied to cells (3.9–21.2 kPa) was three to four digits larger than the stress one magnetic-vortex microdisc provided to a cell (1 Pa) (Kim et al., 2010) which resulted in trauma to the cell membrane and only a few cells underwent apoptosis in our case.

In contrast to the static compression (Cheng et al., 2009), the oscillatory compressive MS in the present study contributes to damaging cancer cells for a shorter time with necrosis as dominant mode of MSICD. Lien, et al. (2013) found that shear stress was effective for autophagy and apoptotic cell death in four different human cancer cells while the effects of oscillating shear stress on cancer cell death are found much less than the static shear stress. The shape of cells in the peripheral region in our study are stretched but not in the central region (Fig. 3B–D). To see the correlation between the radius of cell culture dish and the rate of necrosis mode of MSICD, we extracted the data of MDA-MB-231 (40 µm α was applied for 300 s, where we can observe a force- and time-dependent increase of MSICD) and plotted them in Fig. 5. More MSICD occurred when the distance from the center of the culture dish (r) became larger, especially further than 10 mm. This may support the hypothesis that the cells in the peripheral region are treated with shear stress in addition to the cyclic compression.

Since we observed the dominant mode of MSICD being necrosis in Experiment 1, we also applied small average stress (0.1–1.0 kPa) to cells by choosing the parameters of α=10 µm, time (t)=30, 60, 90 s to give a moderate MS condition in Experiment 2.

Unlike the cells treated with harsh conditions in Experiment 1, two cell lines resulted in more apoptosis (8–10-fold increased) together with necrosis (Fig. 6) in Experiment 2. Loading an external MS with a small α and shorter time seems to be an ideal condition to induce immunogenic cell death because both apoptosis and the release of damage-associated molecular patterns (DAMPs) by necrotic cells are important for the induction of tumor-specific adaptive immune responses (Spel et al., 2013). The early apoptosis rate in Fig. 6 ranged 5–35% for MS loading no longer than 1 min, which is smaller than the late apoptosis data (22–60% positivity) from microdisc-treated cells for 10 min (Kim et al., 2010). However, compared with the study based on static compression stress loading on murine mammary carcinoma cells, where a static compression stress of up to 7.7 kPa (58 mmHg applied to monolayer cells, refer to fig. 7A in Cheng et al., 2009) was applied, our results of dynamic compression stress loading on breast cancer cells in Experiment 2 exhibit higher caspase3/7 activity under the magnitude of the compression stress of 1.0 kPa, being relatively close to the static compression stress of 1.5 kPa (11.6 mmHg). They (Cheng et al., 2009) applied the static compression for 17 h, whereas our case is only for 60 s. This implies that the use of ‘dynamic’ compressive stress loading with even less magnitude of stress than the corresponding static value seems to result in more damage to cells.

In Experiment 2, the applied stress slightly decreased when the frequency became higher, from 0.1 to 30 Hz, e.g. 0.8 to 0.5 kPa, because of the delay in the forcing the piezo-actuator controller at higher frequency, resulting in a net decrease in the applied compression force. This may be a reason why we observed a frequency-dependent decrease of MSICD in mild condition.

We also noticed that two cell lines reacted to the external MS differently. In Experiment 1, BT-474 cells revealed more necrotic cell damage than MDA-MB-231 cells when treated with a longer duration of 300s (Fig. 4).

MDA-MB-231 tends to detach easier from culture flasks/dishes than BT-474 during a routine subculture. MDA-MB-231 and BT-474 cells are metastatic and non-metastatic types, respectively, and molecules that facilitate cancer to cell-extracellular matrix adhesion and movement of tumor cells are expressed higher in MDA-MB-231 than in BT- 474 (Rizwan et al., 2015). Therefore, the damage from the MS accumulated to a lethal level in BT-474 cells while they remained at the original location in culture dishes, which resulted in more necrosis and less apoptosis for a longer duration. Due to the loose attachment to culture dishes, MDA-MB-231 cells may have not experienced cell damage to a lethal level and remained in ‘dying’ status, which we observed as more apoptotic cells after MS for 60 s.

In conclusion, the breast cancer cells underwent cell death with mixed mode of apoptosis and necrosis after dynamic MS was applied within a rather short duration. As for the key parameters we tested, we observed a force- and time-dependent increase of MSICD, while a frequency-dependent change caused a decrease, i.e. MSICD is frequency independent. Table 1 shows a comparison among the past studies and ours on MISCD, summarizing that the dynamic compression stress achieves high rates of MSICD in a shorter time including our data. It is noted in Table 1 that some of the past studies, particularly those based on the spinning nano/micro-sized particles and disc did not provide us with the corresponding shear stress level due to the difficulty of estimating the viscosity of cells and also the effective thin layer thickness which would undergo larger shear-strain rate. In the present study, the necrotic cell damage increased when cells were treated with larger α and for a longer duration, whereas apoptosis increased in MDA-MB-231 at 60 s when they were treated with smaller α. In summarizing the key MS loading parameters (duration time, stress loading level and frequency) on the degree of MSCID and its modes (apoptosis, necrosis and a mixture of them), we can make approximate estimate of the cell damage in terms of the energy loss of a cell under oscillating mechanical stress per cycle and per unit volume expressed by Δ*w* given by Kadooka and Taya (2018) and Taya (2019):(1)
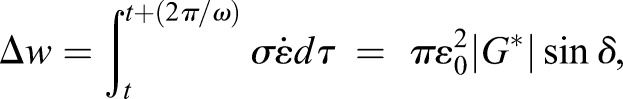
where G* is complex modulus of target cancer cells, *ω* is angular frequency (equal to 2*πf*, *f* is frequency) and G*(*ω*) is given by:(2)
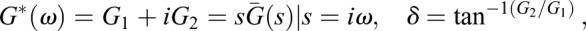
and where *σ*, 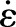 are stress and strain-rate, *ε*_0_ is the strain amplitude, *G*_1_ and *G*_2_ are the real and imaginary part of the complex modulus of G*(*ω*). In Eqn 1, *δ* is the energy-dissipation parameter defined by Eqn 3, sin *δ* is the energy-dissipation term, *τ* is time parameter in integral, *ω* is angular frequency. In Eqn 2, *s* is the Laplace transform parameter that corresponds to time *t* of the original equation, *G̅*(*s*) is the Laplace transform of the relaxation function of *G*(*t*) where setting *s* = *iω*, we can convert the Laplace transform of relaxation function, *G̅*(*s*) to its Fourier transform, G*(*ω*). Based on the crude assumption of the cells made of a viscoelastic model, we can estimate the total energy loss (W) of the cell for the entire duration time, *t*, under applied frequency *f*:(3)
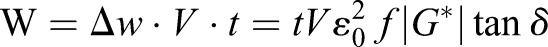

**Table 1.**
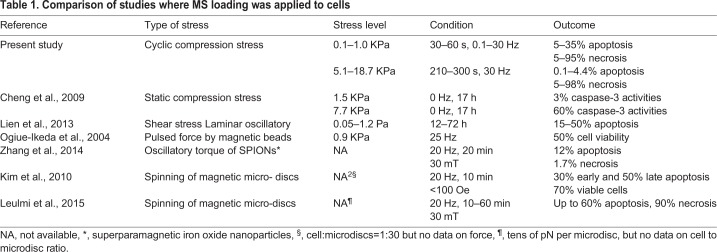
**Comparison of studies where MS loading was applied to cells**

Since the term of *f*|G*|tan* δ* being a non-linear function of frequency *f*, we examined the W-dependence on *f*, *f*|G*|tan *δ* based on the viscoelastic model, the details of which are given in the Supplementary Information, where the key result frequency-dependent terms of *f*|G*|tan *δ* are plotted in Fig. S5, where the cell damage increases from *f*=0 (static mechanical loading) toward its maximum value at *f*=0.21 Hz, then gradually tapers to a constant value as *f* increases further (see Fig. S5). Thus we can conclude that the cell damage is not sensitive to *f* for the majority of the low range of *f* we studied. This prediction of frequency independence of the cell damage is in agreement with our experimental results shown in Fig. 6. Eqn 3 also reveals that W is an increasing function of *t*, and quadratically increases function of displacement loading ε_0_ (or equivalently applies stress in our present experiment). The initial part of the MSICD shown in Fig. 4 in both cells in the central region exhibits a sharp increase with applied stress, seemingly close to quadratic dependence of applied stress (thus equivalently applied displacement loading) although the degree of MSICD under increasing applied stress rapidly reaches the 100% level. The present finding of a mixture of apoptosis and necrosis with necrosis-dominant MSICD has encouraged us, because we aim to damage cancer cells by applying oscillating MS loading based on the flexible nanohelix-nanorobots made of ferromagnetic shape memory alloy, Fe_70_Pd_30_ (Taya et al., 2017) so that the cancer cells release DAMPs due to the necrosis-dominant damaged cells that recruit more immune cells to the cancer site.

## MATERIALS AND METHODS

We tested two breast cancer cell lines, BT-474 and MDA-MB-231 (ATCC, VA, USA), in a series of experiments where the first is luminal B subtype and the second is triple-negative breast cancer, thus very difficult to cure. These cells were cultured in DMEM supplemented with 10% fetal bovine serum (FBS) at 37°C, 5% CO_2_. These cells, with passages of 21–25 times, were seeded in 35 mm culture dishes prior to MS loading testing to reach 2.0×10^4^ cell/cm^2^ before the experiment. We treated the cells in three ways to identify cell damage caused by MS: (1) culture media were moved to DMEM without FBS and placed outside of the CO_2_ incubator during MS loading (Group ‘DMEM’); (2) culture media were moved to DMEM, gently overlaid with an agarose gel (3%, 2 mm thickness) and kept outside of the CO_2_ incubator for the same duration as the third group instead of applying MS (Group ‘w/o MS’); and (3) culture media were moved to DMEM and gel overlay, followed by MS loading testing (Group ‘w/ MS’). Our homemade apparatus to apply dynamic MS onto cells (Fig. 1A) consisted of a piezo actuator and control system (PiezoMove, Physik Instrumente, USA), and the movement of platen and applied force were acquired using the LabVIEW program (National Instruments, USA). After setting cells at the sample holder, the platen was controlled to reach the surface of agarose gel and push cells repeatedly with the desired displacement and cycle numbers (Fig. 1B). The MS loading apparatus provided an oscillating displacement-controlled loading unit which generates the corresponding oscillating mechanical compression force (see Fig. 2). The diameter of Teflon platen was 34.2 mm. To maintain the positive compression force (*F*>0) until the end of each experiment without having an overshooting force at the beginning, the following equation for displacement control loading, *u*(*t*), was integrated in the program:(4)
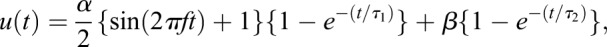
where *α* is displacement amplitude (µm), *f* is displacement frequency (Hz), *t* is time (s), *τ*_1_ is time constant for the ramp-up stage (s), *τ*_2_ is time constant for the additional compressive load (s) and *β* is additional compressive displacement to prevent tensile loading (µm). The platen was manually operated to reach the surface of agarose gels with modest compressive force (∼0.2 N) and then we run the program. We performed two different experiments: Experiment 1 for larger displacement loading with longer duration and Experiment 2 for smaller displacement loading with shorter duration, which are further explained below.

### Experiment 1

We chose 40, 70, 100 and 130 µm as an α to provide the amplitude of force loading, Δ*F*=*F*_max_−*F*_min_, where ΔFs range between 3.6 N and 19.5 N (Fig. 2). Frequency was fixed to 30 Hz. The duration (*t*) was set at 210 and 300 s.

After 4 h of incubation, all specimens were stained with probes of NucBlue, Caspase- 3/7 Green, and Propidium Iodide (PI) (Molecular Probes, USA) and observed under a confocal laser scanning microscope (SP8 TCS, Leica, USA). To avoid underestimating a rate of MSICD, we set the post-MS incubation at 4 h to provide enough time to detect caspase-3/7 signals (Rehm et al., 2002), but not to allow escaped cells recover and proliferate before staining, which may affect the MSICD rate by a larger number of viable cells. Etoposide-treated cells were included in each set of experiments as a positive control of cell damage. The rate of apoptosis or necrosis was calculated by number of caspase-3/7-positive or PI-positive cells/number of NucBlue positive cells×100 (%), respectively.

### Experiment 2

The parameters for the second set of experiments were set to α=10 (µm), *t*=30, 60 and 90 (s), *f*=0.1, 1, 5, 10 and 30 (Hz), where ΔFs ranged between 0.1 N and 1.0 N, and the post- MS incubation time was 4 h. All specimens were stained and observed as described above.

### Statistical analysis

Statistical analysis was performed with two-way ANOVA with Tukey's or Bonferroni's multiple comparisons test and correlation test by using Prism 6 software (Graph Pad, USA). *P*<0.05 was considered significant.

## Supplementary Material

Supplementary information
