# Supplementary material for: Mechanical stress-induced cell death in breast cancer cells
Source: Biol Open. 2019 Aug 7;8(8):bio043133. doi: 10.1242/bio.043133 (PMC6737978; doi:10.1242/bio.043133)
Supplement: Supplementary information [file biolopen-8-043133-s1.pdf]

Biology Open (2019): doi:10.1242/bio.043133: Supplementary information

Supplemental Information on Viscoelastic Model for 3% Agarose Gel and cancer cells (MDA-MB-231) in Compression Test

The basis of viscoelastic model is to obtain the relaxation modulus, (t) of a given material (in this paper, agarose gel) which is placed between the pushing platen and target cancer cells as shown in Fig. 1A. G(t) is defined as the stress relaxation function under applied strain of unit magnitude in Heaviside function form. Experimentally, we cannot apply exactly the ideal unit Heaviside function, thus, we employed a displacement loading with short rise time of 0.3 second. Rise time is defined as the time set for the platen to reach max compressive displacement.

We apply $200\mu m$ compressive displacement (corresponding to 10% strain). Figs. S1(A) and S1(B) show the applied displacement loading of step function type, and the measured force-time relation, respectively.

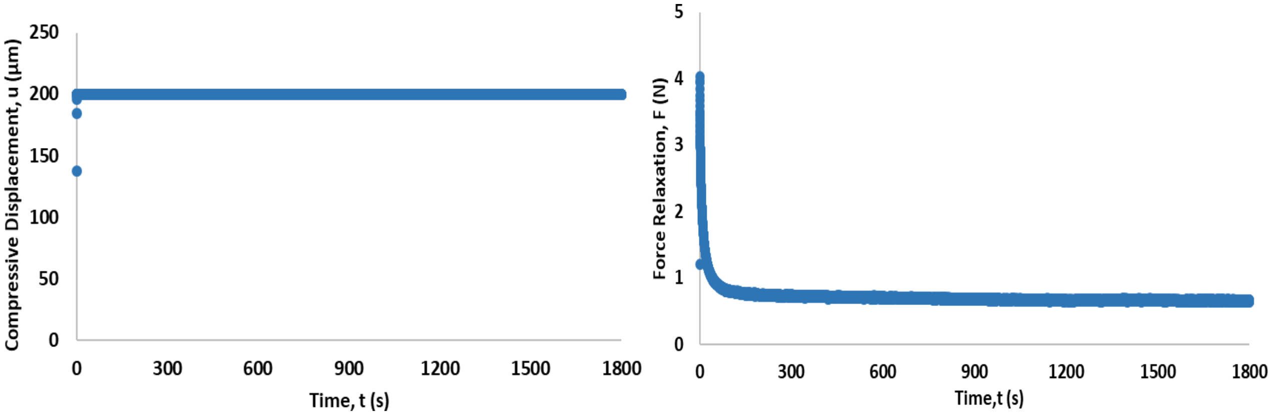

(A) **(B)**

Fig.S1: (A) The applied displacement-loading of step function type with short rise time of 0.3 seconds where the rise time is the time for platen to reach constant displacement (or constant strain), (B) The relaxation force of the 3% Agarose Gel under applied displacement loading of $200um$ with short rise time of 0.3 seconds (10% Strain).

Relaxation functions, (t) the 3% agarose gel obtained experimentally are simulated by the following Prony Series fitting defined by:

$$G(t) = G_o - \sum_{i=1}^{3} G_i \left(1 - e^{-\frac{t}{\tau_i}}\right) \qquad (S1)$$

Where, the parameters in the Prony series, G_0, G_1-G_3 and τ_1-τ_3 will be obtained by curve fitting software of MATLAB by using the experimental data of Figs. S1 (B).

Biology Open • Supplementary information

Hence, we conduct non-linear fitting of the Prony Series, eq. (S1) via MATLAB where G(t) is redefined as the stress relaxation function by diving the force relation function of Fig.

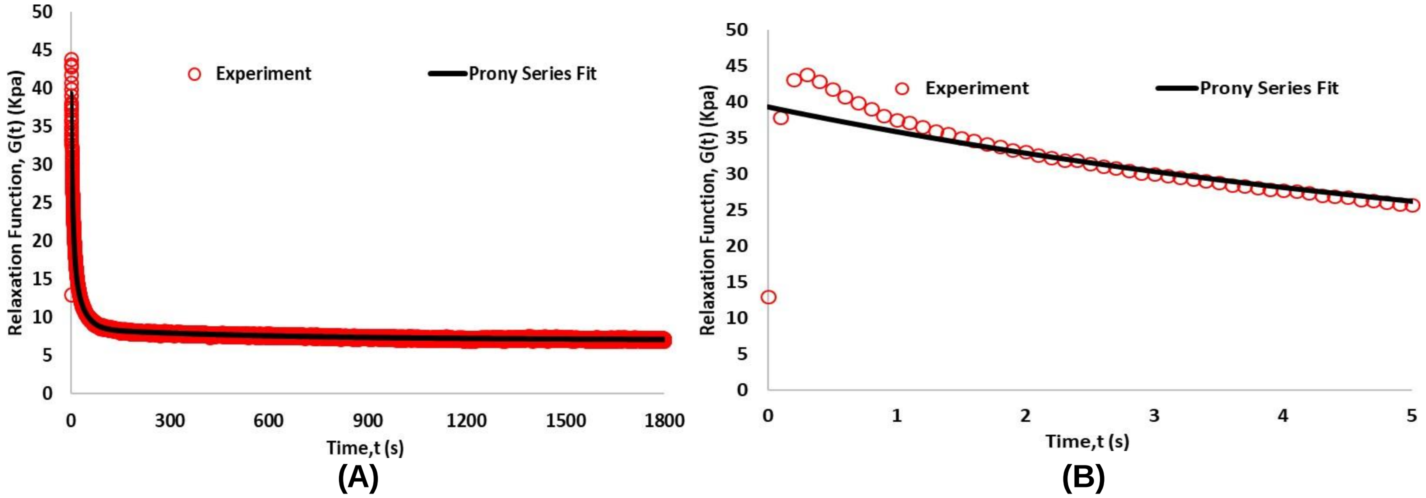

Figure S2: Fitting of Prony Series of the relaxation compressive stress at constant 10% strain for 0.3s rise time where (A) and (B) denote the Prony-series fitted curve of the relaxation function over the entire time without truncation, and zoomed in initial time range, respectively.

S1 (B) by the area of the platen and normalized by strain amplitude which was already obtained from the displacement-loading curve by dividing it by the cross-sectional area of the platen. The results of the Prony series simulation curves of the relaxation functions of short 0.3s rise times are shown in Figs. S2, and the parameters of the Prony series of the stress relaxation function with short 0.3s rise times are given in Tables S1.

Static Disp (um)	ϵ_o,Strain (%)	G_o (KPa)	G_1 (KPa)	G_2 (KPa)	G_3 (KPa)	τ_1 (s)	τ_2 (s)	τ_3 (s)
200	10	39.36	1.48	10.42	20.41	607.12	28.84	6.09

Table S1: The coefficients of Prony Series of the Relaxation function with short rise time under applied displacement loading of 200 um.

Biology Open • Supplementary information

It is clear from Fig. S2 (B) that the Prony-series curve fitting for the initial rise time of 0.3 second does not provide decent agreement with the experimental data. If we remove the initial rise time section (0.3 second) of the relaxation function data of Fig. S2 (A), by setting t=0 at the original initial time of t=0.3 second, we obtain the truncated relaxation curve, see Fig. S3 where (A) shows the truncated relaxation curves (experimental data with red open circles, Prony-series curve fitting with black solid line) over the entire time while (B) for the zoomed initial time frame, indicating better agreement between the experimental data and Prony-series curve fitting compared to those shown in Fig.S3(B).

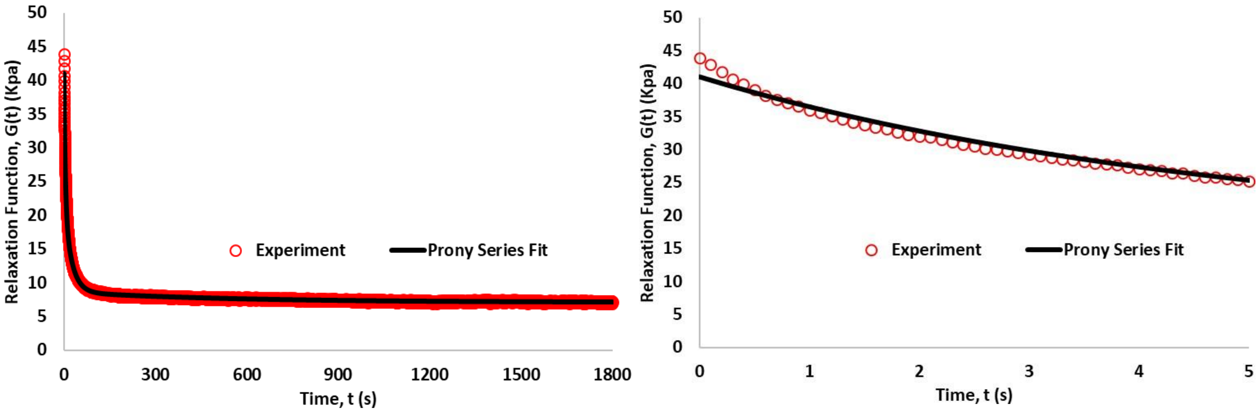

(A) **(B)**

Fig. S3 (A): The Prony Series fitting of the truncated relaxation function for applied displacement loading of 200 um (corresponding to 10% Strain), (B): The zoomed view of figure (S5a) of the truncated relaxation function of 3% agarose gel under applied displacement loading of 200 um for the initial time frame where time=0 corresponds to the original time of 0.3 second.

The corresponding parameter values of the Prony Series fitting of Fig. S4 are listed in the 2nd row of Table S2. In addition, we conducted another Prony Series fitting similar to Fig. S4 but for the case of 300um Static Displacement and the corresponding parameters are tabulated in the 3rd row of Table S2.

Static Disp (um)	ϵ_o, Strain (%)	$G_o\ (KPa)$	$G_1\ (KPa)$	$G_2\ (KPa)$	$G_3\ (KPa)$	$\tau_1\ (s)$	$\tau_2\ (s)$	$\tau_3\ (s)$
200	10	41.03	1.55	13.43	18.97	545.09	23.96	4.24
300	15	143.65	24.02	40.39	44.22	580.51	115.1	11.89

Table S2: The parameters of Prony Series of truncated stress relaxation function of short rise time of 0.3 second under applied displacement loading of 200 um.

Biology Open • Supplementary information

Biology Open (2019): doi:10.1242/bio.043133: Supplementary information

Model to estimate the cell damage underneath 3% agarose gel based on energy loss model of viscoelastic material with complex modulus, G*($j\omega$).

Real (G_1) and imaginary (G_2) parts of a viscoelastic material with complex modulus, G*($j\omega$) (Kadooka and Taya, 2018; Taya, 2019) are defined by

$$\boldsymbol{G_1 = G_R + \omega \int_{0^-}^{\infty} \sin(\omega\tau)\, \widehat{G}(\tau) d\tau} \quad \text{(S2)}$$

$$\boldsymbol{G_2 = \omega \int_{0^-}^{\infty} \cos(\omega\tau)\, \widehat{G}(\tau) d\tau} \quad \text{(S3)}$$

Where, $\widehat{G}(t) = G(t) - G_R$, $G_R = G(t \to \infty)$, $G(t)$ is the Prony series defined by eq.(S1). Hence,

$$G_R = G_o - \sum_{i=1}^{3} G_i \quad \text{(S4)}$$

Applying eq.(S1) to eqs. (S2) and (S3) and performing integrals of exponential functions, we obtain the following G_1 and G_2 in closed form:

$$\boldsymbol{G_1 = G_o - \sum_{i=1}^{3} \frac{G_i + 2G_i\tau_i^2\omega^2}{1+\omega^2\tau_i^2}} \quad \text{(S5)}$$

$$\boldsymbol{G_2 = \omega \sum_{i=1}^{3} \frac{\tau_i G_i}{1+\omega^2\tau_i^2}} \quad \text{(S6)}$$

For the total energy loss (W) of the cells for the entire duration time, t, under applied frequency f, defined by eq. (4) of the main text of this paper, the frequency(f) -dependent energy loss term is given by $f\,|G*|\,tan\delta$ where G*, G_1, G_2 and tan δ are defined by eq. (3). The frequency dependent energy loss term of the cell damage model, $f\,|G*|\,tan\delta$ is calculated using the truncated relaxation function of short rise time of 0.3 sec, the results of which are plotted as a function of f in Fig. S4.

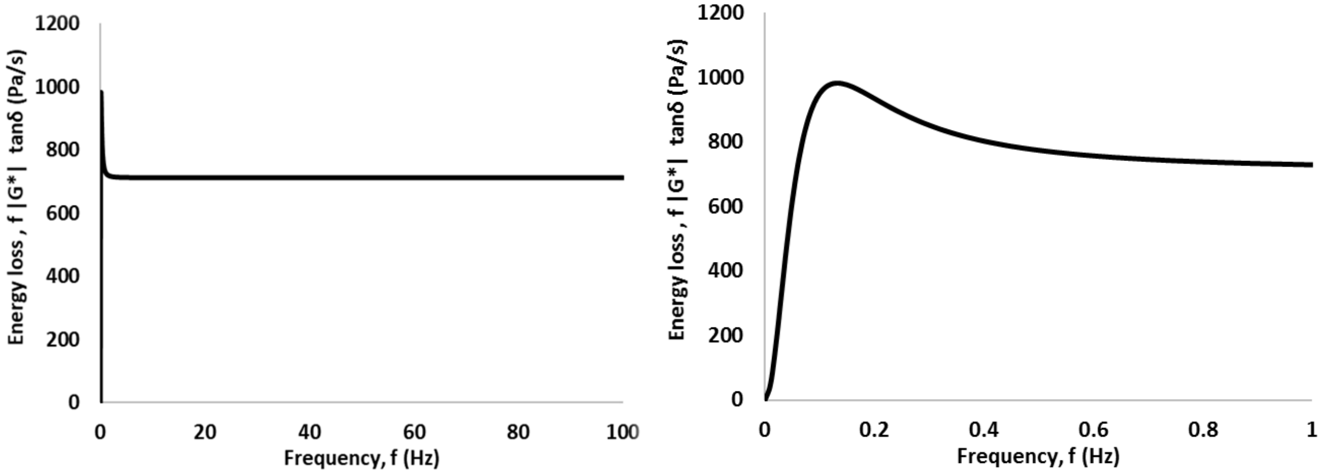

(A) ***(B)***

Biology Open • Supplementary information

Fig. S4 (A): The frequency-dependent energy loss term vs frequency f of 3% agarose gel under applied displacement of 200 um. (B): Frequency-dependent energy loss term of the cell damage model base on the truncated relaxation function of 0.3s rise time under applied displacement loading of 200 um at lower frequency.

It is observed from Figure S4 (A) that there is an energy loss peak at very low frequency and Fig. S4 (B) is the zoomed view of the peak shown in Fig. S4 (A) at the low frequency region. It follows from Fig. S4 that the frequency-dependence of the agarose gel damage becomes rather insensitive to f, except for lower-frequency range of 0.1-0.3 seconds. Even for such lower frequency range, the magnitude of the energy loss term is about the same level as that of higher frequency range, which is nearly constant over the longer frequency range. The above analysis of the energy loss or damage based on viscoelastic model is applied to agarose gel, not cancer cells. In our experiment of applying oscillating compression loading to two types of cancer cell lines (BT-474 and MDA-MB-231), we placed agarose gel layer between the platen and cancer cells, see Fig. 1. Since we did not measure the viscoelastic property of our cancer cell lines, we refer to the work by Efremov et al (2017) who measured the viscoelastic property, particularly the relaxation function based on simplified Prony series (only using the constant and first exponential terms) of selected cell lines (MDA-MB-231 and MCF-7 breast cancer cells). The simplified Prony series is defined in using Eq. (S1) is given by eq. (S7a), below.

$$\boldsymbol{G(t) = G_0 - G_1\left(1 - e^{-\frac{t}{\tau_i}}\right)} \qquad \text{(S7a)}$$

$$\boldsymbol{E(t) = E_\infty + (E_0 - E_\infty)e^{-\alpha t}} \qquad \text{(S7b)}$$

Eq. (S7b) is the expression used by Efremov et al (2017), thus the following inter-relations among those parameters of the simplified Prony series are valid.

$$\boldsymbol{G_0 - G_1 = E_\infty} \qquad \text{(S8a)}$$

$$\boldsymbol{\tau_i = 1/\alpha} \qquad \text{(S8b)}$$

By using the measured data by Efremov et al (2017) on MDA-MB-231 cell type, we obtain the parameters of the simplified Prony series, given in Table S3.

Parameters of simplified Prony series	Data taken from Efremov et al (2017)
$\boldsymbol{E_\infty}$	1 KPa
E_0	2 KPa
α	0.2 /sec
G_1	1 KPa
G_0	2 KPa
τ_1	5 sec.

Table S3 Parameters of simplified Prony series, eq.(S8) based on the cancer data of MDA-MB-231 taken from Efremov et al (2017).

Biology Open • Supplementary information

By using the data of MD-MB-231 and our model of energy loss, eq. (4) of the main text and eqs.(S5)-(S8) of this supplemental information, we obtain the energy loss term which is frequency-dependent, $f\ |G*|\ tan\delta$, the results of which are plotted as a function of frequency f in Fig. S5 where (A) and (B) denote the case of covering the longer range of frequency (f = 0 – 100 Hz) and shorter range (f = 0 – 1 Hz), respectively.

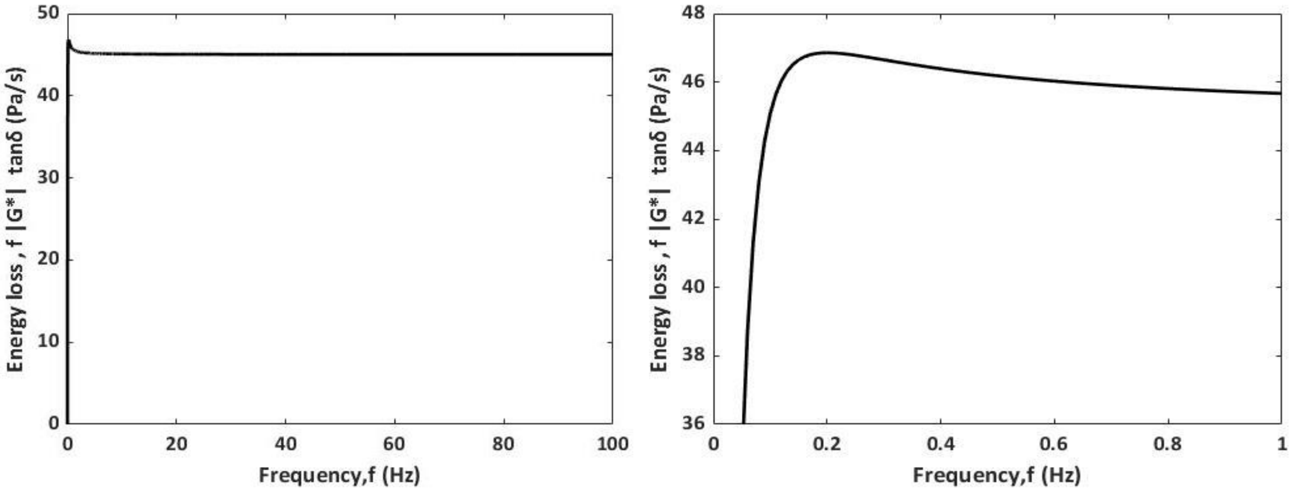

(A) ***(B)***

Fig. S5 Energy loss vs frequency relation of cancer cells (MDA-MB-231) under dynamic mechanical loading (A) for entire frequency range (0 – 100 Hz) and (B) zoomed shorter frequency range (0 – 1 Hz) where the experimental data of the viscoelastic property of the cancer cells with simplified Prony series are taken from Efremov et al (2017) and the energy loss term is defined by eq. (4) and eqs. (S5) and (S6).

A comparison between Figs. S4 and S5, particularly (S4B) and (S5B), reveals that frequency dependence of energy loss term of $f\ |G*|\ tan\delta$ is similar to both agarose gel and cancer cell line of MDA-MB-231, although the magnitude of the agarose gel is much higher than that of the cancer cell line. The results of Fig. S5 indicate that the damage of cancer cells by dynamic mechanical loading has trend of gradually increasing function of f, and peaked at smaller f value near f = 0.21 Hz , thereafter, it becomes almost constant, i.e., frequency-independent.

Biology Open • Supplementary information

References of the supplemental information:

Efremov, Y.M., Wang, W-H, Hardy, S.D., Geahlen, R.L. and Raman, A. (2017), Measuring nanoscale viscoelastic parameters of cells directly from AFM force-displacement curves, Scientific Reports, 7, 1541

Kadooka, K. and Taya, M., (2018),Review on viscoelastic behavior of dielectric polymers and their actuators, *Proc. of SPIE* 10594, EAPAD XX, 105940 M (March , 2018), doi:10.1117/12.2295116.

Taya, M., (2019), Lecture Notes on Viscoelasticity and Plasticity, University of Washington, January 2019. Only available at University Book Store, Seattle, Washington State.
